# Collagen deposition in lung parenchyma driven by depletion of interstitial Lyve-1^+^ macrophages prevents cigarette smoke-induced emphysema and loss of airway function

**DOI:** 10.3389/fimmu.2024.1493395

**Published:** 2025-01-03

**Authors:** Yinebeb Mezgebu Dagnachew, Hwee Ying Lim, Liao Wupeng, Sheau Yng Lim, Sheng Jie Natalie Lim, Chung Hwee Thiam, Shu Wen Tan, Joan Lau Joo Eng, Dan Mei, Syaza Hazwany Mohammad Azhar, Wei Siong Ong, Qi Hui Caris Tan, Wai-Shiu Fred Wong, Veronique Angeli

**Affiliations:** ^1^ Immunology Translational Research Programme, Yong Loo Lin School of Medicine, Department of Microbiology & Immunology, National University of Singapore, Singapore, Singapore; ^2^ Immunology Programme, Life Sciences Institute, National University of Singapore, Singapore, Singapore; ^3^ Department of Biomedical Sciences, School of Medicine, Bahir Dar University, Bahir Dar, Ethiopia; ^4^ Department of Pharmacology, Yong Loo Lin School of Medicine, National University of Singapore, Singapore, Singapore; ^5^ Singapore-Hebrew University of Jerusalem Alliance for Research and Enterprise, National University of Singapore, Singapore, Singapore; ^6^ Drug Discovery and Optimization Platform (DDOP), Yong Loo Lin School of Medicine, National University Health System, Singapore, Singapore

**Keywords:** macrophage, chronic obstructive pulmonary disease, collagen, extracellular matrice (ECM), Lyve-1 macrophage

## Abstract

**Introduction:**

Collagen is essential for maintaining lung structure and function and its remodeling has been associated with respiratory diseases including chronic obstructive pulmonary disease (COPD). However, the cellular mechanisms driving collagen remodeling and the functional implications of this process in the pathophysiology of pulmonary diseases remain poorly understood.

**Methods:**

To address this question, we employed *Lyve1^wt/cre^*; *Csf1r^flox/flox^* mice with specific depletion of Lyve-1^+^ macrophages and assessed the content, types and organization of collagen in lung compartments at steady state and after chronic exposure to cigarette smoke (CS).

**Results:**

Using this mouse model, we found that the absence of this subpopulation of tissue resident macrophage led to the deposition of type I collagen fibers around the alveoli and bronchi at steady state. Further analysis by polarized light microscopy and Sircol collagen assay revealed that the collagen fibers accumulating in the lungs depleted of Lyve-1^+^ macrophages were thicker and crosslinked. A decrease in MMP-9 gene expression and proteolytic activity together with an increase in *Col1a1, Timp-3* and *Lox* expression accompanied the collagen alterations. Next, we investigated the effect of the collagen remodeling on the pathophysiology of COPD and airway function in mice lacking Lyve-1^+^ macrophages exposed chronically to cigarette smoke (CS), a well-established animal model of COPD. We found that deposition of collagen prior CS exposure protected these mice against destruction of alveoli (emphysema), and bronchi thickening and prevented loss of airway function.

**Discussion:**

Thus, we uncover that interstitial Lyve-1^+^ macrophages regulate the composition, amount, and architecture of collagen network in the lungs at steady state and that such collagen remodeling functionally impacts the development of COPD. This study further supports the potential of targeting collagen as promising approaches to treat respiratory diseases.

## Introduction

The lungs are one of the most important vital organs supporting exchange of oxygen and carbon dioxide between the alveoli and the blood capillaries. In addition to their respiratory function, the lungs also serve as an immune organ by hosting immune cells and promoting innate and adaptive immune responses (1). Proteomic and genomic studies identified more than 300 extracellular matrix (ECM) molecules in the lungs that orchestrate directly or indirectly tissue morphogenesis, homeostasis and injury-repair responses in addition to provide a scaffold for cells (2). The lung harbours ECM with a unique composition and action that is essential for its vital functions. Despite the variety of ECM proteins in the lung, collagen and elastin fibers are the major components constituting over 60% (3) and 24% (4) of dry weight of the lung, respectively. Approximately over twenty different collagen subtypes, classified as fibrillar collagen (e.g collagen subtypes I and III) and non-fibrillar collagen (e.g collagen subtype IV) have been identified. The fibrillar collagen is found in the lung parenchyma whereas the non-fibrillar subtype is present in the basement membrane of the lung. Collagen type I is more stiff than collagen type III and hence, the ability of the lung to resist breakage induced by mechanical force during stretching is determined by the ratio of collagen subtype I/III (5). Collagen type I and III confer tensile strength and flexibility, respectively whereas elastin provides recoil properties to the lung. Collagen is a dynamic structure whereby its homeostasis is controlled by the balance between two processes: production and degradation. Collagen production is controlled by genes involved in collagen synthesis such as *Col1a1* and *Col3a1* whereas its degradation is controlled by matrix metalloproteinases (MMPs) and tissue inhibitors of MMPs (TIMPs) (6).

Regulation of ECM remodelling is paramount in maintaining the physiological functions of the lungs. As a consequence, alterations in the amount, distribution and composition of ECM are associated with pathological lung remodelling in multiple respiratory diseases including chronic obstructive pulmonary disease (COPD), asthma and pulmonary arterial hypertension (7). COPD is associated with two distinct anatomic lesions, namely emphysema and small airway remodeling which lead to the progressive reduction in lung function (1). Cigarette smoking is the main causative agent of COPD while dusts, air pollution particles, fumes and biomass fuels are additional identified risk factors (8). In emphysema, inflammatory cells recruited in the alveolar spaces and the airways in response to chronic exposure to cigarette smoke secrete proteolytic enzymes such as elastase and MMPs which degrade elastin and other ECM components. Current concepts of COPD pathogenesis propose that the imbalance between matrix synthesis and degradation during tissue repair may account for either tissue destruction of the parenchyma (emphysema) or fibrosis in airway walls. Despite the evidence for the central role of ECM remodelling in COPD pathophysiology (1, 9), how ECM alterations in the different lung compartment may affect lung function in this chronic pulmonary disease remain unclear.

In the lungs, two populations of macrophages reside in different anatomical compartment, namely: alveolar macrophage (AMs) in the luminal surface of the alveolar space, and the non-alveolar interstitial macrophage (IM). In addition to their differential localization in the lung, these two types of macrophages are phenotypically and functionally different. AMs express Siglec F, high levels of CD11c and low levels of CD11b whereas IMs express high levels of CD11b and low levels of CD11c (10). AMs are important in regulating surfactant turnover, efferocytosis, removal of foreign particles, and defence against inhaled pathogens (11). Recently, we delineated two subpopulations of lung IMs based on the differential expression of major histocompatibility complex (MHC) class II and lymphatic endothelial cell receptor-1 (Lyve-1), Lyve-1^hi^MHCII^lo^ and Lyve-1^lo^MHCII^hi^ (12). Notably, these IMs subpopulations exhibit distinct functions (13). Lyve-1^lo^MHCII^hi^ macrophages regulates immune response whereas Lyve-1^hi^MHCII^lo^ macrophages play a more tissue homeostatic role by restraining induced lung fibrosis (12). This function of lung Lyve-1^hi^MHCII^lo^ macrophages is in agreement with our previous study demonstrating in mice that arterial macrophages expressing Lyve-1 maintain homeostatic vascular tone though the control of collagen via a MMP-9 dependent mechanism (14). Whether lung Lyve-1^hi^MHCII^lo^ (Lyve-1^+^) macrophages can regulate collagen homeostasis in this tissue and impact lung function during respiratory diseases remain to be elucidated. To address these important questions given the roles of collagen in lung structure and function, we used *Lyve1^wt/cre^; Csf1r^flox/flox^* mice with specific depletion of Lyve-1^+^ macrophages and assessed the content, types and organization of collagen in lung compartments at steady state and after chronic exposure to cigarette smoke (CS) to identify a role for pulmonary collagen remodelling in protection against emphysema and loss of airway function in COPD.

## Material and methods

### Animals and COPD model induced by CS chronic exposure

*C57BL/6, Lyve1^cre/cre^ and Csf1r^flox/flox^* were purchased from Jackson Laboratory. *Lyve1^cre/cre^ and Csf1r^flox/flox^* were cross-bred to generate *Lyve1^wt/cre^;Csf1r^flox/flox^* mice (14). All animals were maintained in specific pathogen-free animal facility (Comparative Medicine, National University of Singapore) and were handled in accordance to protocols approved by the institutional animal care and use committee (IACUC) of the National University of Singapore.

Female mice from 15 to 18 weeks of age were used for the experiments. Only female were used in this study because the depletion of Lyve-1^+^ macrophages in lung is mainly observed in female mice. To induce COPD, 15 weeks old female *Lyve1^wt/cre^;Csf1r^flox/flox^* and Csf1r*^flox/flox^* mice were exposed to 4% CS from nine 3R4F reference cigarettes (University of Kentucky, Lexington, KY, USA) daily at a frequency of 3 cigarettes every 2 h for 8 weeks at a frequency of 5 consecutive days per week as described previously (15).

### Flow cytometry

Lung tissues were extracted, minced, and digested for 30 minutes with 0.2mg/ml of collagenase (Sigma). The digested materials were filtered through 160mm nylon mesh filter and pelleted by centrifugation at 1200rpm for 5 minutes at 4°C. Cells were stained at 4°C in FACS buffer containing 1% normal mouse serum (Jackson ImmunoResearch Laboratories).

The following fluorochrome- and biotin-conjugated mouse specific antibodies were used; CD64 (clone X54-5/7.1.1), CD45 (clone 30-F11), LYVE-1 (clone ALY7), CD11b (clone M1/70), CD115 (clone AFS98), F4/80 (clone BM8), MerTK (R&D System), MHCII (clone M5/114.15.2), Ly6C (clone HK1.4), SiglecF (clone E50-2440), and streptavidin PE-CF594 (BD Horizon). Alveolar macrophages were identified as CD45^+^ CD64^+^ MerTK^+^ SiglecF+. Interstitial macrophages were identified as CD45^+^CD64^+^MerTK^+^SiglecF^-^ (12). Lung monocytes were identified as CD45^+^ CD11b^+^ Ly6C^+^. Dead cells were excluded by using DAPI. The cells were acquired on Aurora Spectral Analyser (Cytek Biosciences) and analyzed with Flowjo Software (Tree Star, Inc).

### Sircol soluble collagen assay

Soluble collagen was determined by Sircol soluble collagen assay kit (Biocolor S1000 Kit). Lung tissues were mixed with digestion mix (1M Acetic Acid and 0.2mg/ml Pepsin) and incubated overnight on a rotating Ferris wheel in cold room. On the next day the tubes were centrifuged at 12,000X g for 10 mins. Supernatant was transferred into a new tube. The pellet was kept for insoluble collagen assay (Biocolor S2000 kit). To concentrate collagen in the test sample, 100μl of neutralizing reagent was added to the supernatant and 200 µl of cold isolation and concentration reagent was added to each tube. The content was mixed thoroughly by tube inversions and incubated on metal rack overnight on ice in cold room. Next day tubes were centrifuged at 12,000 rpm for 10 mins. The supernatant was removed, and the pellet was resuspended with 100μl of 0.5M Acetic Acid. Collagen standards with 6 different dilutions (0µl, 20µl, 40µl 60µl, 80 µl & 100 µl) were prepared and topped up by 0.5M acetic acid to a total volume of 100 µl. Equal amount of Sircol Dye Reagent (1ml) was mixed with 20ul test sample and 100µl of prepared collagen reference standard and incubated for 30 mins on vortex mixer. Then the tubes were centrifuged at 12,000rpm for 10 mins. The tubes were inverted carefully to drain the supernatant. Gently 750ul of ice-cold Acid-Salt Wash Reagent was added to the pellet by inverting 3 times. Then the content was centrifuged at 12,000rpm for 10 mins. The supernatant was removed slowly using a pipette then by using a cotton bud. Equal amount of Alkali Reagent (250µl) was added to blanks, standards, and tests samples. Then the tubes were vortexed and shacked on a mechanical shaker for 10 mins. Then 200ul of Sircol dye- alkali reagent from each tube was transferred to a 96 well micro well plate and absorbance was measured at 555nm.

### Sircol insoluble collagen assay

Insoluble collagen was determined using Sircol insoluble collagen assay kit (Biocolor S2000 Kit). The pellet collected from the sircol soluble collagen assay, after pepsin-acetic acid digestion was fragmented by adding Fragmentation Reagent to the pellet (50μl/mg of tissue) and incubating the mixture at 65°C for 2-3 hrs. Then the tubes were centrifuged at 12,000 rpm for 10 mins and the supernatant (test sample) was transferred into fresh tubes. Based on the weight of the tissue, from 5μl or 50μl of test sample was transferred to a new tube for each reaction and topped up to 100μl using dH_2_0. Denatured collagen standards with 5 different dilutions (0µl, 20µl, 40µl 60µl & 100 µl) were prepared and topped up to a total volume of 100 µl using dH_2_0. Then 20 µl of test sample was incubated with 100μl of prepared Collagen Reference Standards & 1ml of Sircol Dye Reagent. The content was mixed by inversion and incubated for 30 mins with mixing. The tubes were centrifuged at 12,000rpm for 10 mins. The supernatant was drained carefully by inverting tubes.

Gently 750μl of ice-cold Acid-Salt Wash Reagent was added to the pellet by inverting 3 times. Then the content was centrifuged at 12,000rpm for 10 mins. The supernatant was removed slowly using a pipette then by using a cotton bud. Equal amount of Alkali Reagent (1ml) was added to blanks, standards, and tests samples. Then the tubes were vortexed and shacked on a mechanical shaker for 10 mins. Then 200ul of Sircol dye- alkali reagent from each tube was transferred to a 96 well micro well plate and absorbance was measured at 555nm.

### Histology

Lung tissues were fixed in 2% paraformaldehyde (PFA) and 30% sucrose and prepared for Cryo-sectioning (10µm). Picrosirus Red (PSR) staining was done using commercially available kit according to manufacturer instruction to estimate the amount/nature of collagen. Bright field microscope was used to take images. Linear polarized microscope was used to see nature of collagen. Cryo sections of the lung (10µm) were also stained with hematoxylin and eosin (H and E) for checking morphology of the lung. The condition of emphysema was assessed by observing the size of alveoli by using the mean linear intercept technique (MLI). MLI refers to the total length of hypothetical line on the lung sections divided by the numbers of alveolar walls intersecting the test lines as described (16). Fields that include larger airways and blood vessels were excluded from the analysis. The analysis was done using 30 different fields of view per lung using the 20X objective. ImageJ software was used to quantify the amount of collagen. Collagen quantification from images taken using bright field microscope was done by converting PSR stained image in to RGB image and by thresholding the collagen stained with only red. The collagen quantification from linear polarized microscope was done by setting 2 Hue values; magenta, red and orange for thick collagen fiber and yellow-green for thin collagen fibers followed by thresholding of each colour.

### Immunohistochemistry

Cryo-sections of mouse lungs (10µm) were prepared for immunohistochemistry by using 2% paraformaldehyde (PFA) and 30% sucrose fixative. Primary antibodies used include anti-LYVE-1 (rabbit polyclonal; Abcam), anti-CD68 (rat clone FA-11; Bio-rad) anti-CD206 (rat clone MR5D3; Bio-rad), anti-CD31 (armenian hamster clone 2H8; Chemicon), anti-collagen type I (rabbit polyclonal; Millipore), and anti-collagen type III (rabbit polyclonal; Abcam).

For isotype controls, rat IgG (eBioscience), armenian hamster IgG (eBioscience), and rabbit IgG (Jackson Immunoresearch) were used. AF488-, Cy3-and AF647- conjugated antibodies from Jackson Immunoresearch were used for detection. FITC or Cy3-conjugated anti smooth muscle actin (SMA, mouse clone 1A4; Sigma-Aldrich) was used to identify smooth muscle cells. Sections were counterstained with 4,6-diamidino-2-phenylindole (DAPI) for cell nuclei visualization and mounted for analysis. Specimens were viewed with a fluorescence widefield (Axio Imager.Z1, Axioxam HRM camera; Carl Zeiss Micro Imaging, Inc., Jena, Germany).

### Quantitative real-time PCR

Immediately after dissection, lung tissues were infused with RNA stabilizing reagent (RNA later, QIAGEN) for one hour at 4 °C degrees. Trizol reagent was used for tissue homogenization and total RNA was extracted from the tissues using an mRNA isolation kit (QIAGEN) according to the manufacturer’s instruction followed by cDNA synthesis from 500-600ng of RNA using Taqman reverse transcription kit (Applied Biosystems). All PCR primers were purchased from Integrated DNA Technologies (Coraville, IA, USA) (listed in **Table 1**). Real time quantitative PCR was performed in triplicates using 100 ng template cDNA in PCR mixture containing SYBR Green Master Mix (Applied Biosystems, Carlsbad, CA, USA). The mRNA expression levels of all samples were normalized with housekeeping gene r12s. Analysis was done using Abi Prism 7500 Detection System (Applied Biosystems, Warrington, UK).

**Table 1:** List of primers used for qPCR.

Gene	Forward primer 5’-3’	Reverse Primer 5’-3’
R12s	GGAAGGCATAGTGCTGGAGGT	CGATGACATCCTTGGCCTGA
Col1a1	ATGTTCAGCTTTGTGGACCT	CAGCTGACTTCAGGGATGT
Col3a1	ATACCCGGAACACGAGGTC	CATCTTCGCCCTTAGGTCCTG
MMP2	TTCAGGTAATAAGCACCCTTGAA	TAACCTGGATGCCGTCGT
MMP9	CGGCACGCCTTGGTGTAGCA	AGGTGAGGGGGCGCCTGTAG
MMP12	TTTGATGAGGCAGAAACGTG	ATCAGCAGAGAGGCGAAATG
Timp1	TCCCCAGAAATCAACGAGACCAC	AGAGTACGCCAGGGAACCAAGAAG
Timp2	CTACACGGCCCCCTCTTCAGCAGT	CAAGGGATCATGGGACAGCGAGTG
Timp3	CAGGGCGCGTGTATGAAGG	CCGGATGCAGGCGTAGTG
Tropoelastin	CGGGTCTGACAGCGGTAGT	CTCCAAGTCCTCCAGGACCT
LOX	CCTGGCCAGTTCAGCATATAG	GTAAGAAGTCCGATGTCCCTTG
LOXL2	GTGAGGGAGACATTCAGAAGAG	GGCACATCGGTGATGTCTAT
TNF-α	CTGTAGCCCACGTCGTAGCAAACC	CGGCTGACGGTGTGGGTGAG
IL-1β	CTTCAAATCTCGCAGCAGCACATC	CCAGCAGGTTATCATCATCATCC
IL-4	AGGTCACAGGAGAAGGGACGCC	TGCGAAGCACCTTGGAAGCCC
IL-10	GCTCTTACTGACTGGCATGAG	CGCAGCTCTAGGAGCATGTG

### Gelatin zymography

The activity of gelatinase enzyme was determined by gelatin zymography assay. Lung tissues were homogenized using tissue homogenizing buffer and protein concentration of the sample was determined by BCA assay kit. Resolving gel with 10% polyacrylamide containing 1% gelatin and stacking gel (5% polyacrylamide) was prepared. Gel electrophoresis was done using 10ug protein without any reducing agent. Then, the gel was washed in renaturing solution. The gel was incubated in development buffer for 15 minutes with gentle agitation at room temperature then transferred to 37°C incubator for 16-20 hours incubation. The gel was stained in staining solution for 4 hours and destained in destaining solution until the bands were visible against blue background of the gel. Image was taken using gel imager (Bio-Rad) and quantification of the gel band intensity which is equivalent to the activity of the enzyme was done using ImageJ (version 1.43).

### Measurement of lung function

Mice were anesthetized with ketamine/medetomidine (75 mg/kg and 1 mg/kg, respectively) 24 h after the last CS exposure, and tracheotomy was performed as described (15). Mice were placed in a whole-body plethysmograph connected to a computer-controlled ventilator (Forced pulmonary maneuver system, Buxco Research System, Wilmington, NC, USA) (17). Functional residual capacity (FRC), static compliance (C_chord_), forced expiratory volume at 100ms over forced vital capacity (FEV100/FVC), FEV at 50ms over FVC (FEV50/FVC) and FEV at 20ms over FVC (FEV20/FVC) and total lung capacity (TLC) were recorded using the FinePointe™ data acquisition and analysis software (Buxco). The volume of air remaining in the lung after passive expiration is called FRC. If the alveoli are abnormally expanded and unable to contract sufficiently to remove air, large volume of air will be trapped inside the alveoli which increases the value of FRC as observed in the case of emphysema. This may happen due to breakage of elastin (18) and/or presence of less collagen in the parenchyma (19). VC is the volume of air that can be blown out forcefully after maximum inspiration with slow manoeuvre. FVC is the same as VC except the manoeuvre during FVC is rapid and fast. Less VC and FVC values indicate poor lung performance. FEV100 is the volume of air that can forcibly be blown out in first 100ms after full inspiration.

### Statistical analysis

Statistical analysis was performed with Graphpad Prism version 5.0 (GraphPad Software). Data were presented as mean ± SEM and were analysed by nonparametric Mann-Whitney U and significance levels were set at p<0.05.

## Results

### Lyve-1 expressing macrophages reside in murine lung parenchyma

**Figure 1:**
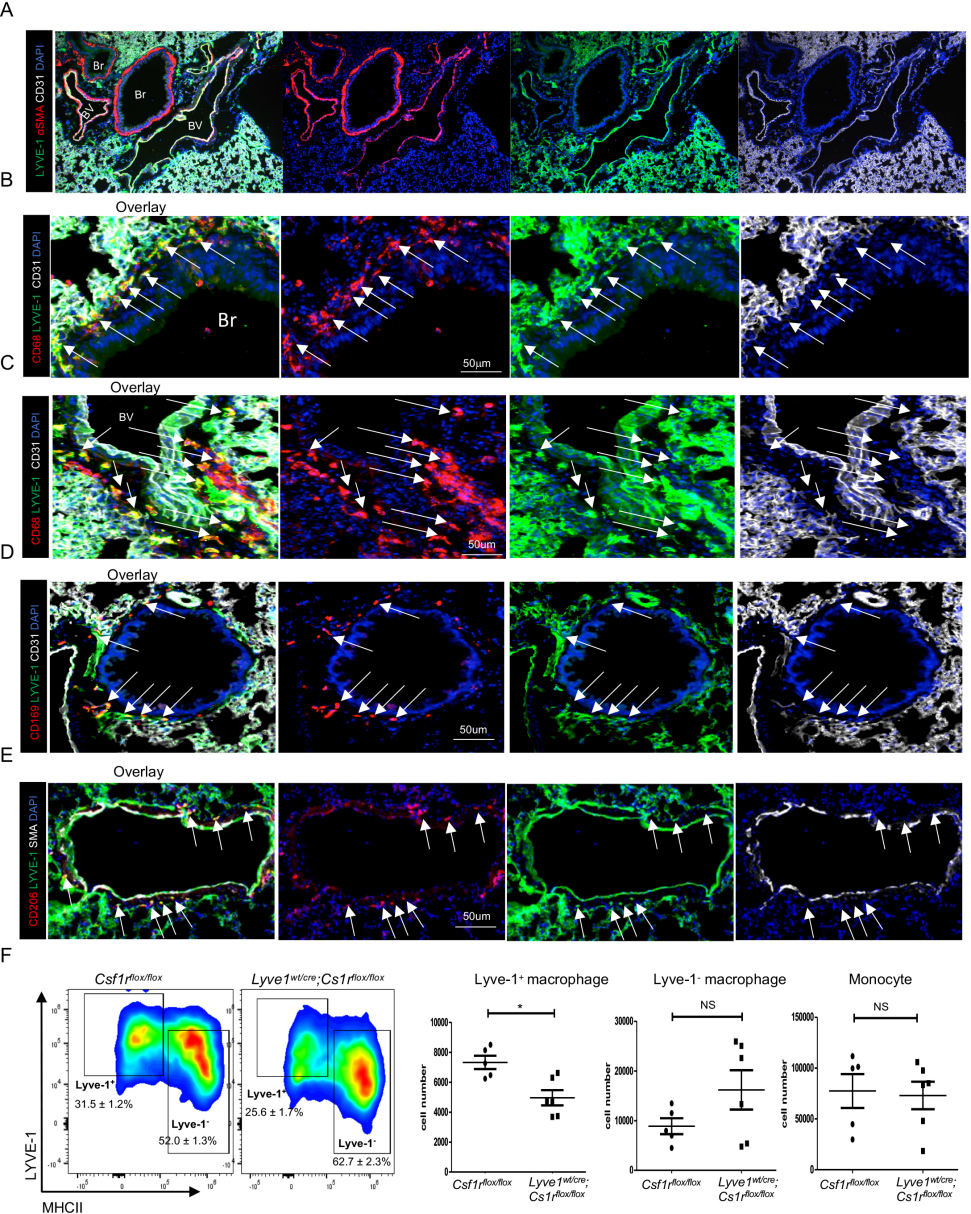
Lyve-1 expressing macrophages reside in murine lung parenchyma. **(A–E)** Immunofluorescence micrographs of lung sections from WT C57BL/6 mice stained for LYVE-1, α-SMA (smooth muscle actin) or CD31, CD68 and CD169 or CD206. Blood vessels (BV) were LYVE-1^+^ SMA^+^ CD31^+^ while bronchioles (Br) were LYVE-1^-^ SMA^+^ CD31. Macrophage were CD68^+^. White arrow indicates LYVE-1^+^ macrophages. Lyve-1^+^ macrophages were found around blood vessel. Lyve-1^+^ macrophages also expressed CD169 and CD206. **(F)** Lyve-1^+^ and Lyve-1^-^macrophages and monocytes number were determined by flow cytometry (mean ± SEM, n = 5-6) in lungs from *Csf1r^flox/flox^ and Lyve-1^wt/cre^;Cs1r^flox/flox^ mice*. *p<0.05.

Consistent with our previous study (12), SiglecF^+^ AMs and SiglecF^-^ IMs were identified in female mouse lungs by flow cytometry analysis at steady state (**Supplementary Figure 1A**). Among the SiglecF^-^ IMs, a sub-population expressed Lyve-1 and their presence in the lungs was further confirmed by immunohistochemistry (**Supplementary Figure 1B**; **Figure 1A**). Since the lung is unique concerning Lyve-1 expression whereby all the reticuloendothelial systems including lymphatic and blood vessels of the lung are positive for Lyve-1, it makes difficult to distinguish different vessel types based on CD31, smooth muscle actin (SMA) and Lyve-1 expression (**Figure 1A**) (12). Thus, we focused our analysis on the larger bronchioles known to exhibit an artery and vein adjacent to it. Blood vessels expressed CD31, a pan-endothelial marker, SMA, and Lyve-1 whereas bronchioles are CD31^-^SMA^+^Lyve-1^-^ (**Figure 1A**). Macrophage were identified by CD68, a pan-macrophage marker. Our immunofluorescent staining showed that Lyve-1^+^ macrophages were observed around the bronchiole and blood vessel (**Figures 1B, C**) and also expressed CD169 (**Figure 1D**). In line with our previous findings showing the M2-like phenotype of tissue Lyve-1^+^ macrophages (12, 14), we found in this study that lung Lyve-1^+^ macrophages were also positive for the M2 macrophage marker CD206 (**Figure 1E**). Next, we analyzed the frequency and number of Lyve-1^+^ macrophages in lung from control *Csf1r^flox/flox^* mice and *Lyve1^wt/cre^; Csf1r^flox/flox^* mice in which Csf1r was specifically depleted in all Lyve-1-expressing cells. Because Lyve-1^+^ macrophages depend on the CSF-1R pathway, Lyve-1^+^ macrophages are depleted in *Lyve1^wt/cre^; Csf1r^flox/flox^* mice. Similar to the aortas (14), depletion of Lyve-1^+^ macrophages was observed in the lungs of *Lyve1^wt/cre^; Csf1r^flox/flox^* mice whereas lung Lyve-1^-^ macrophages and monocytes were not affected (**Figure 1F**).

### Depletion of interstitial Lyve-1^+^ macrophage at steady state leads to collagen deposition in lung parenchyma

Using *Lyve1^wt/cre^; Csf1r^flox/flox^* mice, we investigated how the depletion of Lyve-1^+^ macrophages affects the distribution, content and types of collagen in the lungs at steady state.

**Figure 2:**
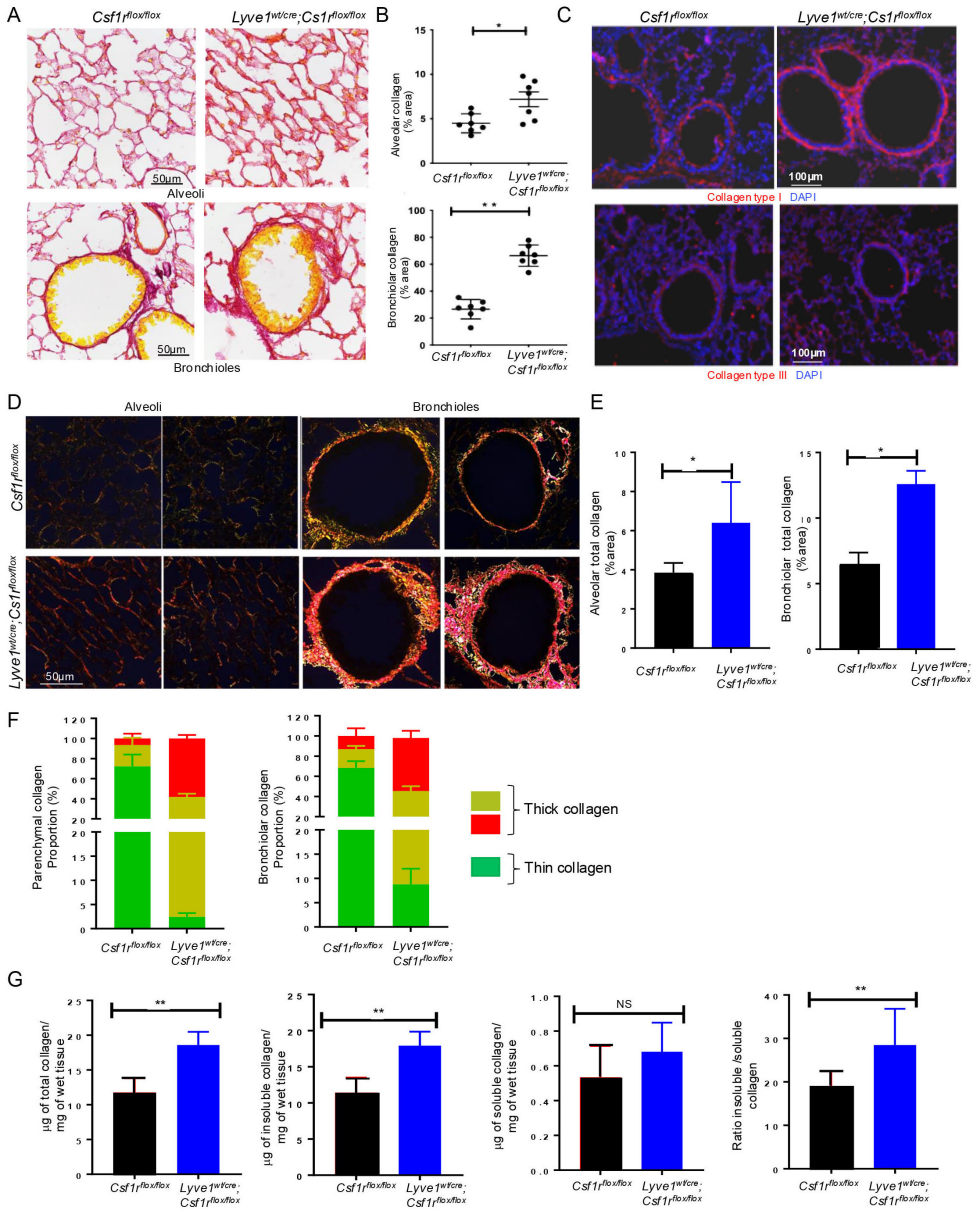
Depletion of Lyve-1^+^ macrophages increases collagen amount in lung parenchyma. **(A)** Lung sections stained for collagen by picrosirius red staining, showing more red (collagen) around the alveoli (top panel) and the bronchioles (bottom panel) in *Lyve-1^wt/cre^; Csf1r^flox/flox^* mice. **(B)** ImageJ quantification of collagen (mean ± SEM, n = 7). **(C)** Immunofluorescence staining of collagen type I and type III in lungs from *Csf1r^flox/flox^* and *Lyve-1^wt/cre^; Csf1r^flox/flox^* mice. **(D)** Photomicrographs of lung tissue sections from *Csf1r^flox/flox^* and *Lyve-1^wt/cre^; Csf1r^flox/flox^* mice stained with picrosirius red and analysed with linear polarized light microscope. **(E)** ImageJ quantification of alveolar and bronchiolar collagen. **(F)** ImageJ quantification of thin and thick collagen fibers around alveoli and bronchioles. **(G)** Sircol collagen assay of lung tissue homogenates from *Csf1r^flox/flox^ and Lyve-1^wt/cre^; Csf1r^flox/flox^* mice. Data collected from 2-3 independent experiments and expressed mean ± SEM; n=5-7 per group. *p<0.05, **p<0.005.

Analysis of lung sections stained with picrosirus red under bright field microscope revealed that collagen content increased both around the alveoli and the bronchioles in *Lyve1^wt/cre^*; *Csf1r^flox/flox^* mice compared to control *Csf1r^flox/flox^* mice (**Figures 2A, B**). To determine which type of collagen contributed the most to this overall increase in lung collagen, we specifically immunostained for collagen type I and type III, the predominant types of collagen in the lung. Notably, collagen type I expression was significantly higher in *Lyve1^wt/cre^*; *Csf1r^flox/flox^* mice compared to control *Csf1r^flox/flox^* mice whereas no difference in the amount of collagen type III was detected (**Figure 2C**).

Under polarized light microscope, the architecture including thickness and organization (orientation and spatial distribution) of collagen fibers stained by picrosirius red is readily identified by the changes of colors and packing density (20). The immature thin collagen fibers have weak birefringence and appear green while the matured thick collagen fibers become orange or red (20). Moreover, immature or less crosslinked collagen is weaker and more susceptible to rupture unlike the mature crosslinked collagen which is relatively stiffer (21, 22). Our polarized light microscopic analysis confirmed the accumulation of alveolar and bronchiolar collagen and revealed a predominant red orange (RO) birefringence, i.e., thick collagen fibers around both the alveoli and the bronchioles of *Lyve*1*^wt/cre^*; *Csf1r^flox/flox^* mice and thinner collagen fibers with yellow green (YG) birefringence in control mice (**Figures 2D, E**). Color quantification of the image confirmed the increased proportion of red/orange collagen in *Lyve1^wt/cre^; Csf1r^flox/flox^* mice compared to control *Csf1r^flox/flox^* mice (**Figure 2F**). This result suggests that the collagen accumulating in *Lyve1^wt/cre^; Csf1r^flox/flox^* mice may be more crosslinked.

To further support the increase in collagen content and predominance of more crosslinked fibers in lungs from *Lyve1^wt/cre^; Csf1r^flox/flox^* mice, we used sircol collagen assay to quantify the amount of soluble and insoluble collagen. Because the solubility of collagen is determined in part by the degree and type of cross-linking between collagen molecules, the soluble collagen is in general less cross-linked, while the insoluble collagen is considered to be more extensively cross-linked (20, 22). The amount of total collagen (the sum of soluble and insoluble collagen), insoluble collagen and the ratio of insoluble to soluble collagen were significantly augmented in lungs of *Lyve1^wt/cre^; Csf1r^flox/flox^* mice than in control mice (**Figure 2G**). Altogether, these data revealed that Lyve-1^+^ macrophages are important in restraining collagen deposition in the lung parenchyma at steady state.

### Remodeling of collagen fiber induced by depletion of Lyve-1^+^ macrophages is associated with reduced MMP-9 proteolytic activity

**Figure 3:**
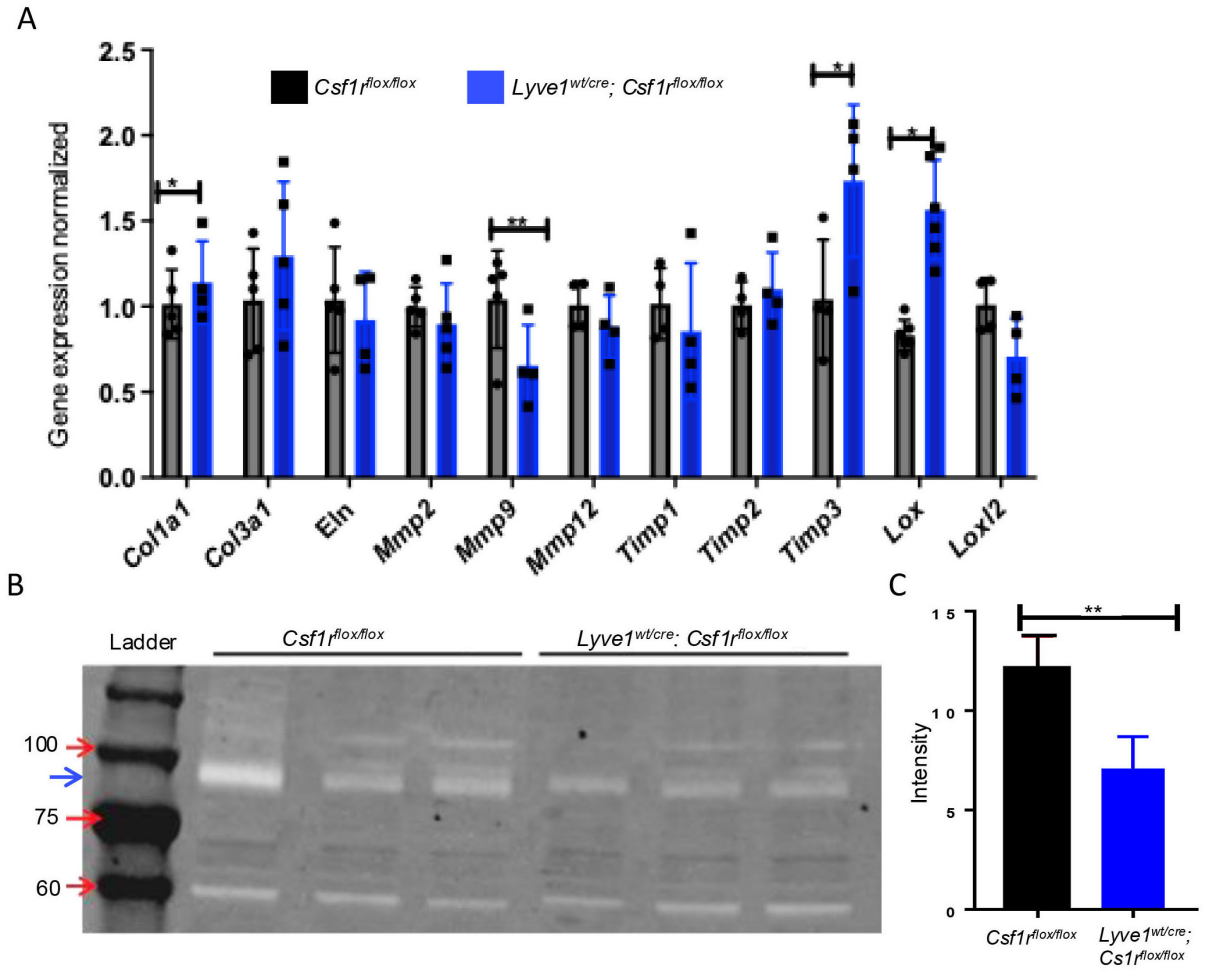
Depletion of Lyve-1^+^ macrophages reduces MMP-9 gene expression and proteolytic activity in lung. **(A)** Relative gene expression analysis of lung tissues *Csf1r^flox/flox^* and *Lyve-1^wt/cre^; Csf1r^flox/flox^* mice by real-time PCR. **(B)** Detection of MMP-9 activity by gelatin zymography. The electrophoretic position of the 82-kDa of MMP-9 activated form is indicated by a blue arrow **(C)** Quantification of MMP-9 activity by measuring band intensity. Data collected from 2-3 independent experiments and expressed mean ± SEM; n=3-5 per group. *p<0.05, **p<0.005.

To understand the mechanism underlying the accumulation of collagen in lungs after depletion of Lyve-1^+^ macrophages, we examined the expression of genes controlling the synthesis and degradation of collagens by real-time PCR. Pro-collagen type 1 alpha 1 (*Col1a1*) gene expression was markedly upregulated in lungs of *Lyve1^wt/cre^; Csf1r^flox/flox^* mice. No significant difference in the mRNA expression levels of tropo-elastin (*Eln*), the precursor of elastin, was observed (**Figure 3A**). Having shown the presence of more thick and crosslinked type of collagen in the lungs of *Lyve1^wt/cre^; Csf1r^flox/flox^* mice, we examined the expression of genes involved in collagen crosslinking. The mRNA level of *Lox* was significantly upregulated in *Lyve1^wt/cre^; Csf1r^flox/flox^* mice while no difference in the expression level of *Loxl2* was noted and *Loxl1* expression was not detected (**Figure 3A**). The expression level of collagen-degrading matrix metalloproteinase 9 (MMP-9) was also significantly decreased in *Lyve1^wt/cre^; Csf1r^flox/flox^* mice (**Figure 3A**). In contrast, the pro-collagen form, MMP inhibitor *Timp3* was upregulated in *Lyve1^wt/cre^; Csf1r^flox/flox^* mice (**Figure 3A**). In addition, gelatin-based zymography assay revealed a reduced MMP-9 proteolytic activity in lung homogenate from *Lyve1^wt/cre^; Csf1r^flox/flox^* mice compared to control mice (**Figure 3B**) which may contribute together with the increased *Col1a1* expression to the accumulation of collagen observed in these mice.

### Changes in collagen in mice depleted of Lyve-1^+^ macrophage does not affect lung function at steady state

**Figure 4:**
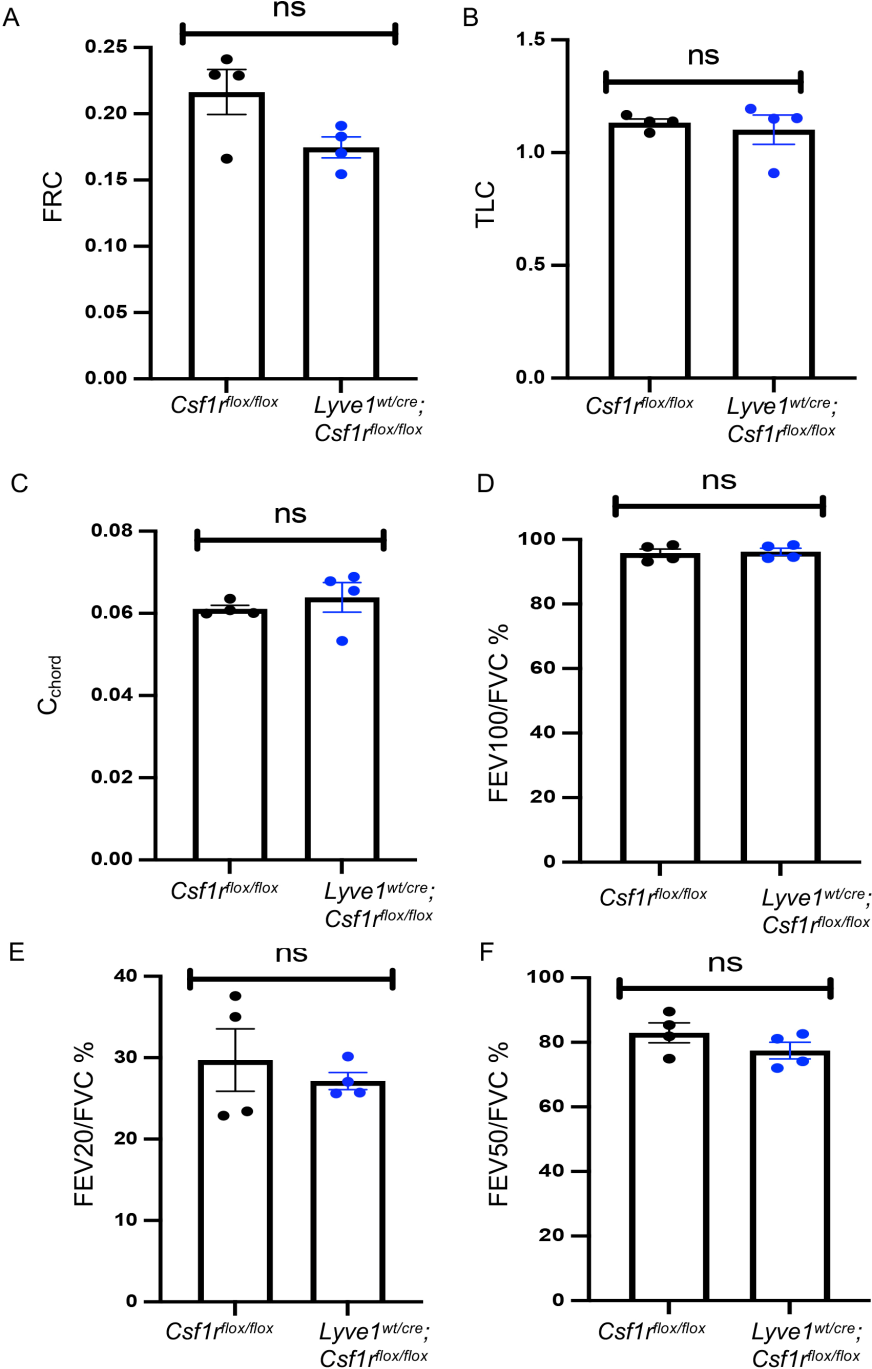
Depletion of Lyve-1^+^ macrophages does not affect lung function at steady state. **(A)** Functional residual capacity (FRC). **(B)** Total lung capacity (TLC). **(C)** static compliance (Cchord). **(D)** Forced expiratory volume (FEV) at 100ms over forced vital capacity (FVC) ratio (FEV100/FVC). **(E)** FEV at 20ms over FVC ratio. **(F)** FEV at 50ms over FVC ratio were measured by plethysmography in *Csf1r^flox/flox^* and *Lyve-1^wt/cre^; Csf1r^flox/flox^* mice exposed to CS (CS) or not (non-CS). Data collected from 2 independent experiments and expressed mean ± SEM; n=5 per group.

Since collagen is an important structural protein which protects the lung from any mechanical stress, we investigated whether the accumulation of collagen observed in the lungs of Lyve1*^wt/cre^*; Csf1r*^flox/flox^* mice impact the homeostatic lung function by conducting lung functional tests at steady state. Lung function examination is a group of tests that enable us to evaluate lung competencies. It includes functional residual capacity (FRC), total lung capacity (TLC), static compliance (Cchord), Forced expiratory volume (FEV) at 100ms over forced vital capacity (FVC) ratio (FEV100/FVC), FEV at 20ms over FVC ratio and FEV at 50ms over FVC ratio. The lung function tests conducted in *Lyve1^wt/cre^; Csf1r^flox/flox^* mice and *Csf1r^flox/flox^* control mice at steady state revealed no difference in all the lung function parameters tested among the two groups (**Figure 4**). These results suggest that the deposition of collagen observed in the lung from mice depleted of Lyve-1^+^ macrophage was not sufficient to alter overall airway function at steady state.

### Depletion of Lyve-1^+^ macrophages prevents emphysema and bronchial thickening in cigarette smoke-induced COPD

**Figure 5:**
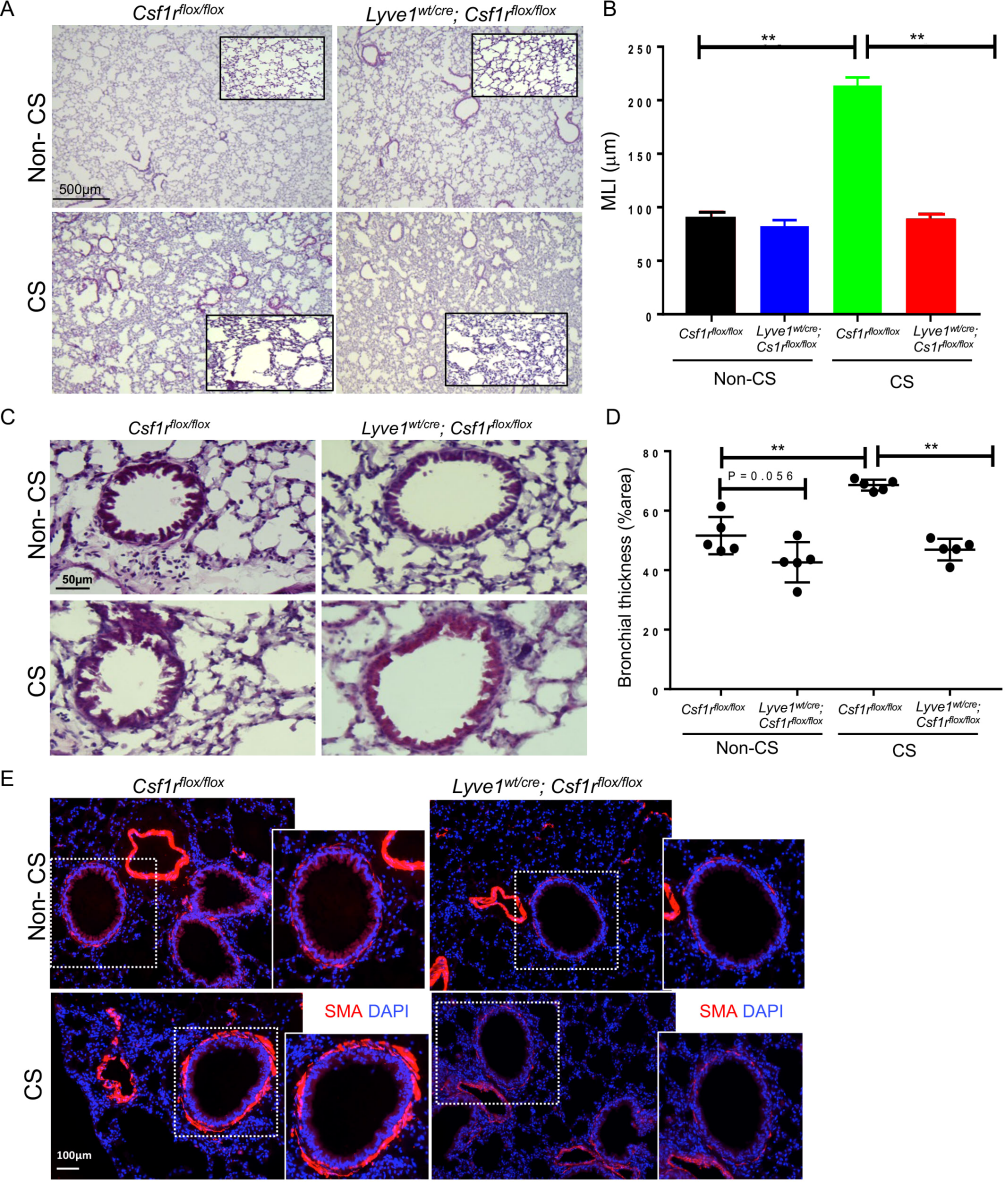
Depletion of LYVE-1^+^ macrophages reduces emphysema and bronchial thickness in CS-induced COPD. **(A)** Representative photomicrographs of H & E-stained lung sections from *Csf1r^flox/flox^ and Lyve-1^wt/cre^; Csf1r^flox/flox^* mice exposed to CS (CS) or not (non-CS). **(B)** Mean linera intercept (MLI) quantification by ImageJ in Csf1rflox/flox and Lyve-1wt/cre; Csf1rflox/flox mice exposed to CS (CS) or not (non-CS). **(C)** Representative photomicrographs of bronchioles on H & E-stained lung sections from *Csf1r^flox/flox^ and Lyve-1^wt/cre^; Csf1r^flox/flox^* mice exposed to CS (CS) or not (non-CS). **(D)** Bronchial thickening quantification by ImageJ in *Csf1r^flox/flox^ and Lyve-1^wt/cre^; Csf1r^flox/flox^* mice exposed to CS (CS) or not (non-CS). **(E)** Representative images of immunofluorescent staining for αSMA of lung sections from *Csf1r^flox/flox^ and Lyve-1^wt/cre^; Csf1r^flox/flox^* mice exposed to CS (CS) or not (non-CS). Data collected from 2-3 independent experiments and expressed mean ± SEM; n=5 per group. **p<0.005.

We sought to investigate the impact of collagen deposition resulting from the depletion of Lyve-1^+^ macrophages on COPD. COPD was induced in *Lyve1^wt/cre^; Csf1r^flox/flox^* and *Csf1r^flox/flox^* control mice through chronic exposure to CS which is a well-established animal model mimicking most of the COPD symptoms seen in human patients (15, 23). No significant differences in mouse weights were detected between *Lyve1^wt/cre^; Csf1r^flox/flox^* and *Csf1r^flox/flox^* control mice after exposure to CS (**Supplementary Figure 2**). As observed at steady state (**Figure 2**), collagen accumulated more in the parenchyma and small airways of *Lyve1^wt/cre^; Csf1r^flox/flox^* mice after CS compared to CS-exposed *Csf1r^flox/flox^* mice (**Supplementary Figure 3**). CS inhalation induces various key features of COPD pathophysiology including emphysema, thickening of bronchial wall, and bronchitis (23). Emphysema, which is characterized by enlargement of the alveoli and, overtime, the rupture of the alveolar wall which ultimately leads to shortness of breath, can be assessed by estimating the size of the alveoli using mean linear intercept method (MLI). The smaller the MLI value is, the larger the size of alveoli is, which designates emphysema (16). Assessment of MLI from lung sections stained with Haematoxylin and Eosin showed emphysema in lungs of *Csf1r^flox/flox^* mice after CS exposure compared to non-CS exposed *Csf1r^flox/flox^* mice (**Figures 5A, B**). In contrast, emphysema was significantly reduced in *Lyve1^wt/cre^; Csf1r^flox/flox^* mice after CS exposure (**Figures 5A, B**). Next, we evaluated the impact of Lyve-1^+^ macrophage depletion on bronchial thickening, another pathophysiological feature of COPD. Haematoxylin and Eosin staining of lung sections revealed that thickening of small airways in *Lyve1^wt/cre^; Csf1r^flox/flox^* mice after CS exposure was also decreased compared to control *Csf1r^flox/flox^* mice (**Figures 5C, D**). COPD is associated with an increase in airway smooth muscle cell mass (23) and bronchial thickening in CS exposed mice is accompanied by thickening of smooth muscle layer in the bronchiole (16). Immunofluorescence staining of SMA that identifies bronchiole smooth muscle cells revealed a marked decreased in SMA expression in bronchioles from *Lyve1^wt/cre^; Csf1r^flox/flox^* mice after CS exposure compared to control *Csf1r^flox/flox^* mice (**Figure 5E**). Finally, we analysed inflammation after CS exposure since it is also associated with COPD. However, the expression of inflammatory genes including *Tnfa*, *Il1b*, *Il10* and *Il4* was not different in *Lyve1^wt/cre^; Csf1r^flox/flox^* mice exposed to CS compared to control *Csf1r^flox/flox^* mice (**Supplementary Figure 4A**). Similarly, no differences were observed for the infiltration of monocytes (**Supplementary Figure 4B**) or macrophages (**Supplementary Figures 4C, D**) between *Lyve1^wt/cre^; Csf1r^flox/flox^* mice and *Csf1r^flox/flox^* mice exposed to CS. These results suggest that depletion of Lyve-1^+^ macrophage prior COPD induction which is accompanied by collagen accumulation mainly restrained emphysema and bronchial thickening without affecting inflammation in response to chronic exposure to CS.

### Depletion of Lyve-1^+^ macrophage prevents loss of lung function after chronic exposure of cigarette smoke

**Figure 6:**
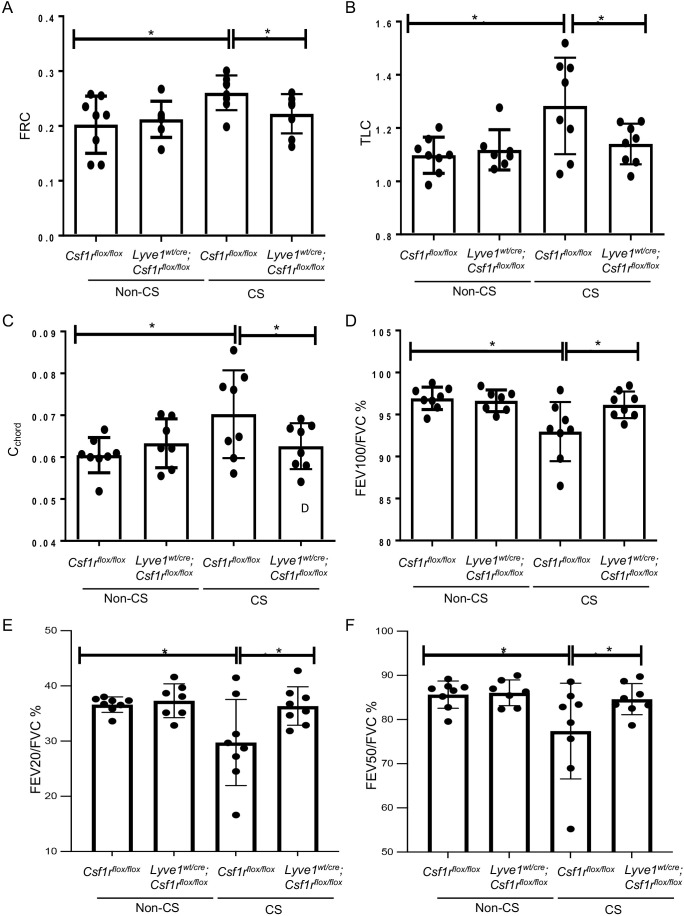
Depletion of Lyve-1 macrophages prevents loss of airway function in CS-induced COPD. **(A)** Functional residual capacity (FRC). **(B)** Total lung capacity (TLC). **(C)** static compliance (Cchord). **(D)** Forced expiratory volume (FEV) at 100ms over forced vital capacity (FVC) ratio (FEV100/FVC). **(E)** FEV at 20ms over FVC ratio. **(F)** FEV at 50ms over FVC ratio were measured by plethysmography in *Csf1r^flox/flox^ and Lyve-1^wt/cre^; Csf1r^flox/flox^* mice exposed to CS (CS) or not (non-CS). Data were collected from 2 independent experiments and expressed mean ± SEM; n=8 per group. *p<0.05.

We investigated whether the reduction in CS-induced emphysema and small airway thickening observed in mice after depletion in Lyve-1^+^ macrophage was sufficient to improve lung function. CS-induced COPD mouse model is characterized by alterations in some lung function parameters including an increase in FRC, TLC and c_chord_ and a decrease in FEV100/FVC, FEV50/FVC and FEV20/FVC. As expected FRC, TLC, and C_chord_ were increased in *Csf1r^flox/flox^* mice after chronic exposure to CS compared to non-CS exposed *Csf1r^flox/flox^* mice (**Figures 6A–C**). However, these lung parameters were improved with depletion of Lyve-1^+^ macrophage (**Figures 6A–C**). Similarly, FEV100/FVC, FEV50/FVC and FEV20/FVC values were decreased in *Csf1r^flox/flox^* mice after CS inhalation, but this decrease was attenuated in *Lyve-1^wt/cre^; Csf1r^flox/flox^* mice (**Figure 6D**). Altogether, lung function tests revealed that depletion in Lyve-1^+^ macrophage overall prevents loss of airway flow in response to chronic exposure to CS.

## Discussion

Here we identify a role for lung collagen remodeling in preventing emphysema and loss of airway function in CS-induced COPD that is controlled by Lyve-1^+^ IMs. Our results extend the collagen regulatory function of Lyve-1^+^ macrophage beyond the arteries (14) by demonstrating that lung resident Lyve-1^+^ macrophages tune the amount, composition and architecture of pulmonary collagen fibers in steady state. Using our mouse model depleting specifically Lyve-1^+^ macrophages, *Lyve1^wt/cre^; Csf1r^flox/flox^* mice, we showed that the reduction of these macrophages leads to the deposition of collagen, especially collagen type I, around the alveolar parenchyma and bronchiole under homeostatic conditions. Further analysis of the thickness and organization of the collagen fibers under polarized light microscopy revealed that the collagen fibers accumulating in the lungs of these mice are thicker and more crosslinked. These results were further supported by the increase in insoluble collagen. Importantly, thicker and more crosslinked collagen fibers are known to be more stable and resistant to degradation through MMPs proteolysis. Notably, the alterations in the collagen fibers of *Lyve1^wt/cre^; Csf1r^flox/flox^* mice were associated with changes in the expression of collagen-related genes controlling either its synthesis/degradation or crosslinking. We found an upregulation in *Col1a1 and Timp-3* expression but a downregulation in *Mmp9* expression that was accompanied by a decreased MMP-9 gelatinase activity in the lungs from *Lyve1^wt/cre^; Csf1r^flox/flox^* mice. The decreased expression of *Mmp9* in lungs of *Lyve1^wt/cre^; Csf1r^flox/flox^* mice is consistent with our previous study in which we reported that arterial Lyve-1^+^ macrophages control collagen deposition by vascular smooth muscle cells via a MMP-9 dependent mechanism (14). However, the increased expression of *Timp-3* and *Lox* in absence of Lyve-1^+^ macrophages suggest additional mechanisms by which these macrophages may regulate collagen content and organization. Since *Timp3* and *Lox* genes were not identified in gene signatures of lung Lyve-1^+^ macrophages (12), but are expressed by lung fibroblasts and smooth muscle cells (24–27), Lyve-1^+^ macrophages could indirectly influence the expression of *Timp3* and *Lox* in these cells. An increase in Timp3 activity will further contribute to the accumulation of collagen observed in absence of Lyve-1^+^ macrophages by inhibiting MMP-dependent collagen degradation while increased Lox activity will contribute to collagen crosslinking and structural stability (28). Notably, mice lacking *Timp3* develop a spontaneous enlargement of air space in the lung associated with reduced collagen abundance and increase collagen degradation (29).

Remodeling of ECM, especially elastin and collagen, has been involved in the pathophysiology of several respiratory diseases including COPD which is a serious global health problem with increasing morbidity, mortality, and economic burden. In the COPD lungs, loss of airway function is associated with the increased degradation of elastin which leads to the fragility of alveoli and ultimately their destruction (30). Although elastase activity is augmented in emphysema (31), there are evidence that elastase and elastin degradation may not represent the only pathway involved in the development of emphysema. For example, emphysema can also be induced by genetic manipulations that increased collagenase activity (31). Moreover, collagenase activity has been shown to be increased in emphysema (32) and proposed to be essential for its development through the weakening of the collagen fibers and loss of mechanical forces (33). A study using second harmonic generation and multiphoton microscopy demonstrated extensive alterations in the elastin and collagen fiber structures in the alveolar region of emphysematous human lung samples (34, 35). Altogether, these findings suggest that preventing the degradation of collagen or promoting its deposition and/or crosslinking may be a protective mechanism in emphysema. Studies on collagen content in COPD have reported conflicting results (36). Increased total collagen content has been detected in lungs of patients with emphysema but expression levels of *Col1a1* and *Col3a1* were shown to be decreased in the lungs of COPD patients and associated with a reduction in FEV1 (37). Consistent with the latter study, Hogg et al. (38), demonstrated a decline in total collagen in bronchioles of severe COPD patients associated with a decrease in type-I/-III ratio. In addition, the study by Annoni et al. (39), reported less collagen type I in the airways of mild/moderate COPD patients, potentially supporting the collapse of airway. Our data also support the potential protective role of collagen remodeling in COPD since the accumulation of more crosslinked collagen, especially type I, in lung prevents emphysema and bronchi thickness in CS-induced COPD mice depleted of Lyve-1^+^ macrophage.

Motivated by the emerging role of ECM in COPD (40), we sought to take advantage of our *Lyve1^wt/cre^; Csf1r^flox/flox^* mouse model that exhibits collagen remodeling in lung parenchyma and bronchi to elucidate the functional implications of collagen changes in lungs. Despite the obvious remodeling of collagen fibers in the lungs induced after the depletion of Lyve-1^+^ macrophages at steady state, these changes were not significant enough to modify the homeostatic lung function parameters in *Lyve1^wt/cre^; Csf1r^flox/flox^* animals. This is not totally unexpected as lung function does not always reflect the pulmonary histological changes (41). In contrast, the accumulation of collagen fibers resulting from Lyve-1^+^ macrophage depletion prevented the loss of lung function in response to chronic CS exposure. Our results provide evidence that quantitative and structural alterations of collagen fibers in lung parenchyma may have a beneficial effect on lung function in COPD by preventing the destruction of lung in parenchymal regions and limiting bronchial thickening. However, our current mouse model does not allow to determine the specific contribution of collagen remodeling in lung parenchyma and small airway to the improvement of lung function parameters. Moreover, our observation that depletion of Lyve-1^+^ macrophages did not affect lung functions at steady state in spite of collagen remodeling suggests that the absence of these macrophages could trigger a compensatory mechanism that might be blunted in response to CS exposure.

In sum, our study uncovers the ability of tissue resident Lyve-1^+^ macrophage in regulating collagen network in the lungs at steady state. Moreover, it unveils the impact of lung collagen remodeling on the pathophysiology of COPD and airway function which may ultimately provide an exciting opportunity to develop novel approaches targeting ECM elements to advance our ability to reduce mortality caused by COPD.

## Data Availability

The original contributions presented in the study are included in the article/**Supplementary Material**. Further inquiries can be directed to the corresponding author.

## Supplementary material

Supplementary Figure 1
**(A)** Gating strategy for lung macrophage populations in C57BL/6 mice. Lung cells were stained for Dapi, CD45, CD11b, MerTK, CD64, Siglec F, Lyve-1 and MHCII and analyzed by flow cytometry. Representative flow cytometric analysis showing identification of SiglecF^+^ alveolar macrophages and SiglecF^-^ interstitial macrophages among MerTK^+^CD64^+^ cells and Lyve-1^hi^MHC^lo^ (Lyve-1^+^) and Lyve-1^lo^MHC^hi^ (Lyve-1-) macrophages among SiglecF^-^ macrophages. **(B)** Representative flow cytometric plot showing identification of alveolar and interstitial macrophage subsets. Number denotes percentage of macrophage subsets (mean ± SEM, n = 5-6).

Supplementary Figure 2 Measurement of mouse weight. Weight of *Csf1r^flox/flox^* and *Lyve-1^wt/cre^*; *Csf1r^flox/flox^* mice exposed to CS (CS) or not (non-CS) was measured before exposure to CS and every week thereafter for 8 weeks and expressed as % relative to initial weight.

Supplementary Figure 3 Depletion of Lyve-1^+^ macrophages induces collagen accumulation in CS-induced COPD. **(A)** Representative images of picrosirus red staining around the alveoli (left panels) and bronchioles (right panels) of *Csf1r^flox/flox^* and *Lyve-1^wt/cre^*; *Cs1r^flox/flox^* mice exposed (CS) or not to CS (non-CS). **(B)** ImageJ quantification of collagen around the bronchiole and in the parenchyma of Csf1rflox/flox and *Lyve-1^wt/cre^*; *Cs1r^flox/flox^* mice exposed (CS) or not to CS (non-CS). Data collected from 2-3 independent experiments and expressed mean ± SEM; n=5-7 mice. *p<0.05; **p<0.005.

Supplementary Figure 4 Depletion of Lyve-1 macrophages does not affect inflammation associated with CS-induced COPD. **(A)** Inflammatory gene expression analyzed by quantitative real time PcR in lungs from *Csf1r^flox/flox^* and *Lyve-1^wt/cre^*; *Cs1r^flox/flox^* mice exposed (CS) or not to CS (non-CS) **(B)** Number of lung monocytes determined by flow cytometry. **(C)** Lung sections stained for CD68 pan macrophage marker. **(D)** Number of lung macrophages determined by flow cytometry. Data collected from 2-3 independent experiments and expressed mean ± SEM; n=5 per group. *p<0.05; **p<0.005.
