# Supplementary figures and images for: Collagen deposition in lung parenchyma driven by depletion of interstitial Lyve-1+ macrophages prevents cigarette smoke-induced emphysema and loss of airway function
Source: Front Immunol. 2025 Jan 3;15:1493395. doi: 10.3389/fimmu.2024.1493395 (PMC11738928; doi:10.3389/fimmu.2024.1493395)

A

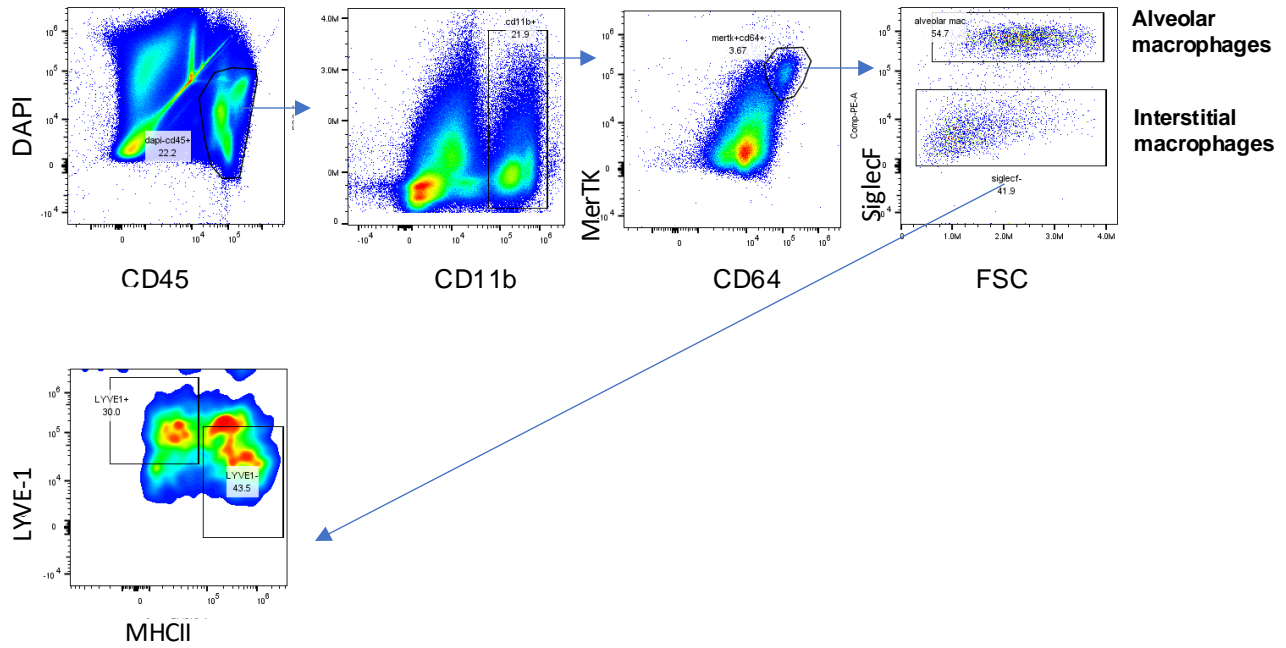

B

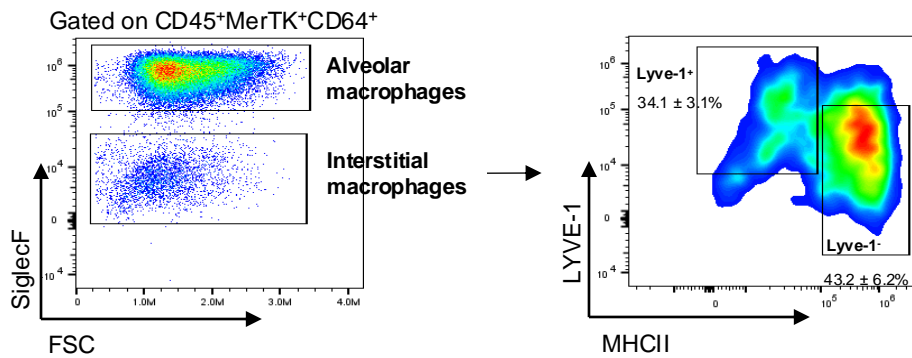

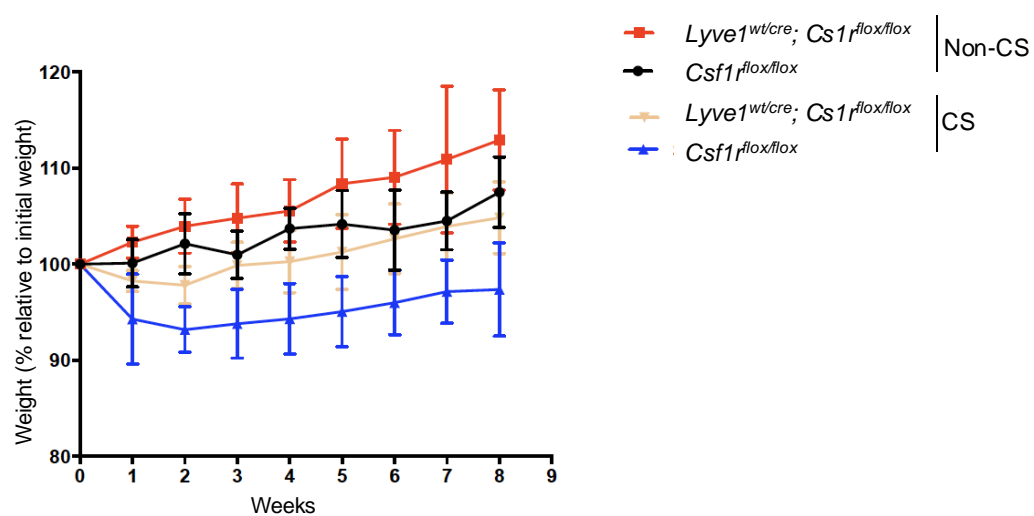

A

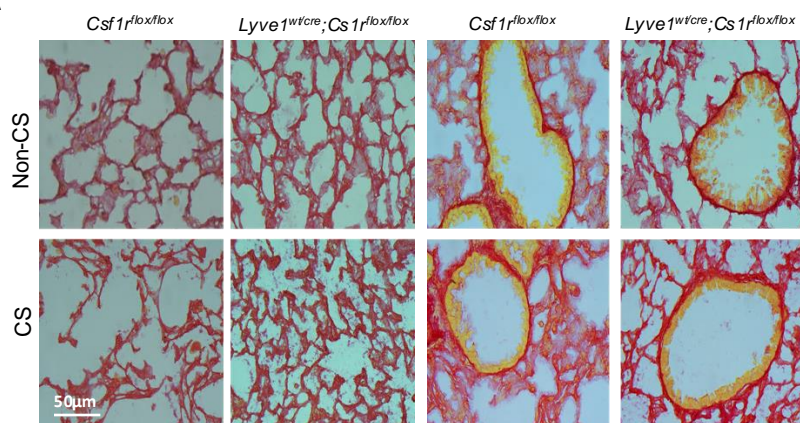

B

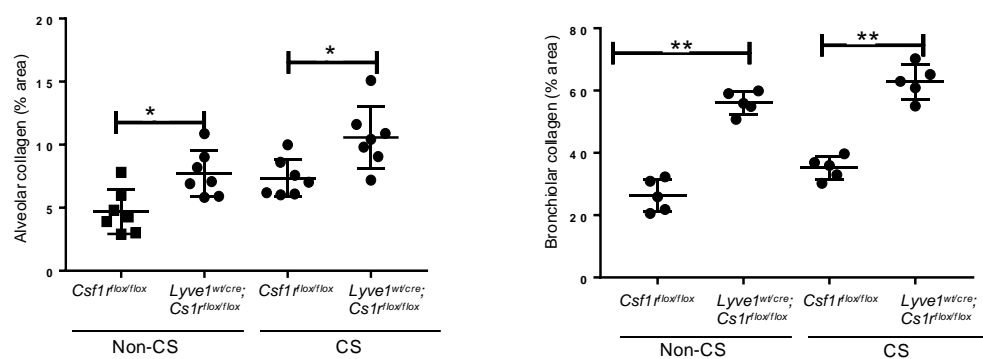

A

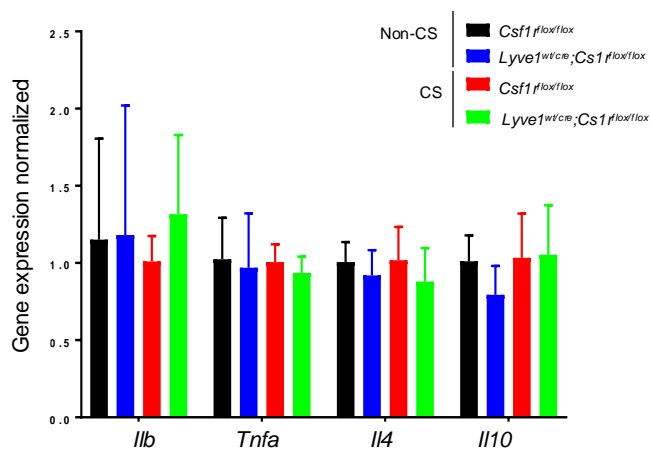

B

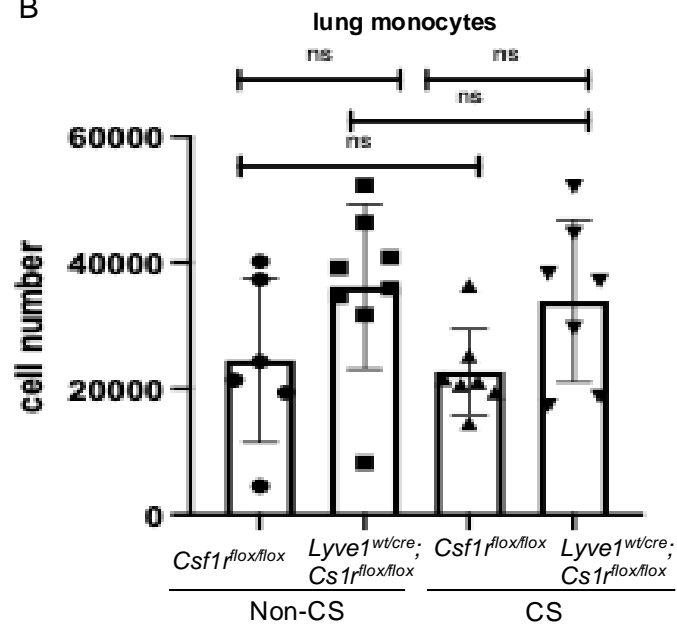

C

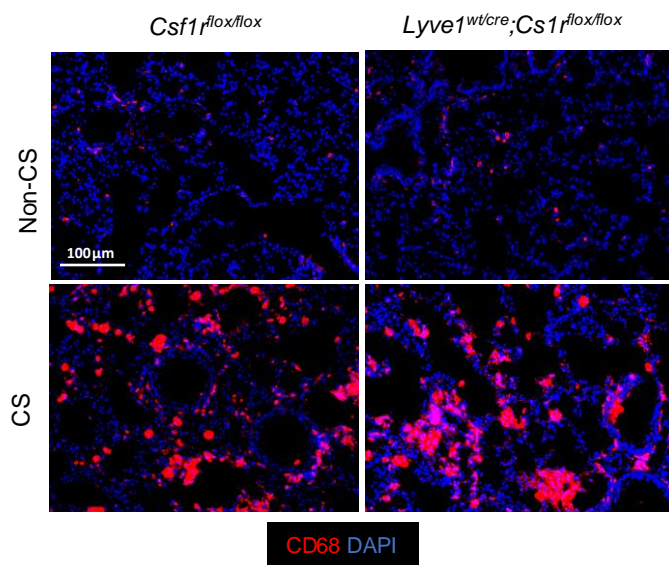

D

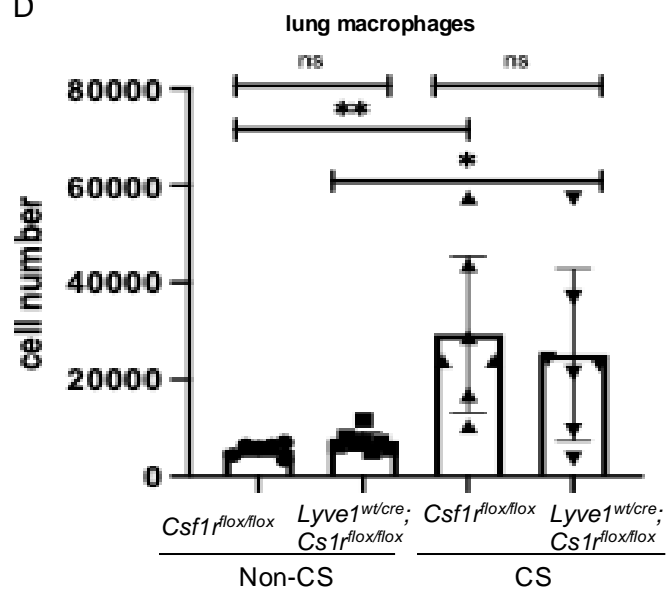

Supplement: Supplementary Figure 1 — (A) Gating strategy for lung macrophage populations in C57BL/6 mice. Lung cells were stained for Dapi, CD45, CD11b, MerTK, CD64, Siglec F, Lyve-1 and MHCII and analyzed by flow cytometry. Representative flow cytometric analysis showing identification of SiglecF+ alveolar macrophages and SiglecF- interstitial macrophages among MerTK+CD64+ cells and Lyve-1hiMHClo (Lyve-1+) and Lyve-1loMHChi (Lyve-1-) macrophages among SiglecF- macrophages. (B) Representative flow cytometric plot showing identification of alveolar and interstitial macrophage subsets. Number denotes percentage of macrophage subsets (mean ± SEM, n = 5-6). [file DataSheet1.pdf]
